# Screening mutations in myosin binding protein C3 gene in a cohort of patients with Hypertrophic Cardiomyopathy

**DOI:** 10.1186/1471-2350-11-67

**Published:** 2010-04-30

**Authors:** María Isabel Rodríguez-García, Lorenzo Monserrat, Martín Ortiz, Xusto Fernández, Laura Cazón, Lucía Núñez, Roberto Barriales-Villa, Emilia Maneiro, Elena Veira, Alfonso Castro-Beiras, Manuel Hermida-Prieto

**Affiliations:** 1 Hospital Universitario de A Coruña-Servicio Galego de Saúde (SERGAS), Instituto de Ciencias de la Salud, As Xubias s/n, A Coruña, 15006, Spain; 2 Instituto de Investigación Biomédica de la Universidad de A Coruña (INIBIC), Instituto de Ciencias de la Salud, As Xubias s/n, A Coruña, 15006, Spain

## Abstract

**Background:**

*MyBPC3 *mutations are amongst the most frequent causes of hypertrophic cardiomyopathy, however, its prevalence varies between populations. They have been associated with mild and late onset disease expression. Our objectives were to establish the prevalence of *MyBPC3 *mutations and determine their associated clinical characteristics in our patients.

**Methods:**

Screening by Single Strand Conformation Polymorphisms (SSCP) and sequencing of the fragments with abnormal motility of the *MyBPC3 *gene in 130 unrelated consecutive HCM index cases. Genotype-Phenotype correlation studies were done in positive families.

**Results:**

16 mutations were found in 20 index cases (15%): 5 novel [D75N, V471E, Q327fs, IVS6+5G>A (homozygous), and IVS11-9G>A] and 11 previously described [A216T, R495W, R502Q (2 families), E542Q (3 families), T957S, R1022P (2 families), E1179K, K504del, K600fs, P955fs and IVS29+5G>A]. Maximum wall thickness and age at time of diagnosis were similar to patients with *MYH7 *mutations [25(7) vs. 27(8), p = 0.16], [46(16) vs. 44(19), p = 0.9].

**Conclusions:**

Mutations in *MyBPC3 *are present in 15% of our hypertrophic cardiomyopathy families. Severe hypertrophy and early expression are compatible with the presence of *MyBPC3 *mutations. The genetic diagnosis not only allows avoiding clinical follow up of non carriers but it opens new possibilities that includes: to take preventive clinical decisions in mutation carriers than have not developed the disease yet, the establishment of genotype-phenotype relationship, and to establish a genetic diagnosis routine in patients with familial HCM.

## Background

Hypertrophic Cardiomyopathy (HCM) is an autosomal dominant disorder, characterized by unexplained left ventricular hypertrophy, myocyte hypertrophy and disarray, and interstitial fibrosis [1,2]. It has a frequency of 0.2% in the adult population and is a major cause of sudden cardiac death (SD) in young people (< 35 years old). Ever since the first mutation in the beta myosin heavy chain (*MYH7*) gene was described as a cause of hypertrophic cardiomyopathy (HCM) in 1990 [3], mutations have been identified in 11 genes that codify cardiac sarcomeric proteins [4-7]. Genes that more frequently show mutations are *MYH7 *and the cardiac myosin binding protein C (*MyBPC3*) on chromosomes 14 and 11, respectively. Mutations in *MyBPC3 *are responsible for 15-20% of cases of familial HCM. Besides, available clinical and familial data are very scarce, making it very difficult to confirm the pathogenicity of the described mutations and to establish reliable correlations between genotype and phenotype.

Three isoforms of myosin binding protein-C are known to exist in adult muscle. The three of them have, 10 globular domains termed C1-C10, 7 of which are immunoglobulin I-like (IgI-like) domains, with the remaining three being fibronectin 3 (Fn3) domains. A conserved linker, termed the S2-binding motif, exists between domains C1 and C2. There is also a proline/alanine-rich extension N-terminal of C1. The cardiac isoform has an additional IgI-like domain at the N-terminus (termed C0), an amino acid sequence LAGGGRRIS within the S2-binding motif, and a 28-amino acid insertion within the C5 domain [8,9]. These domains allow their interaction with other sarcomeric proteins.

The objectives of this study were first, determining the frequency and type of mutations in the *MyBPC3 *gene within a range of patients with HMC previously studied for the *MYH7 *[10] gene; second, describing the clinical features of the carriers; and third, analyzing the correlation between genotype and phenotype in the identified mutations.

## Methods

### (i) Patients

One hundred and thirty unrelated consecutive index cases from Complejo Hospitalario Universitario A Coruña, Spain, diagnosed with HCM according to the criteria of the European Society of Cardiology Working Group on Myocardial and Pericardial Diseases [2]. All the patients had been previously studied for mutations in *MYH7 *[10]. We did a prospective follow-up which included the search for the patient's personal and family history, symptoms, physical exploration, electrocardiogram, echocardiogram, ergometry, Holter, treatments and events. The family members were invited to a check-up that included a clinical study, electrocardiogram, echocardiogram and genetic study. The clinical characteristics of this cohort had been previously described [10]. All patients and family members signed an informed consent agreement and the study was approved by the "Comité ético de investigación de Galicia". The study protocol conforms to the ethical guidelines of the 2008 Declaration of Helsinki.

### (ii) Genetic Study

Genomic DNA was extracted from blood anticoagulated with EDTA with the NUCLEON HT Genomic DNA Extraction Kit (Amersham Biosciences, UK). The primers were designed using reference sequence GenBank:U91629.1[11]. The whole codifying sequence and the flanking intronic regions of the *MyBPC3 *gene were amplified. Genetic screening was carried out through chain reaction single-strand conformation polymorphism analysis (SSCP) of each fragment using commercial polyacrylamide gels 15/24 (T = 15%, C = 2%-GeneGel-Amersham Biosciences, UK). Each fragment was run to pH 8.3 and pH 9.0. The temperature was optimized for each pH and each fragment. Fragments with abnormal motility were sequenced using automatic sequencer CEQ 8000 Genetic Analysis System (Beckman Coulter, USA). To test the sensitivity of SSCP, the direct sequence analysis of the exon 12 was done for all samples and no false negative results were detected.

A variant was considered a mutation in accordance with three criteria: cosegregation with affected members in the family, absence of the mutation in 200 healthy adult controls, and the conservation of the mutated residue among species.

Moreover, the index cases were studied using Sequenom MassArray™ system where 537 genetic variants of HCM disease genes (*TNNT2, TNNI3, TPM1, MYL2, MYL3, ACTC, TTN, MYH6, MYLK2, MYO6, TCAP*) were detected by means of MALDI-TOF mass spectrometry after I-PLEX Gold assay (Sequenom Inc.).

### (iii) *In silico *tools

#### Splice Site Score Predictions

Programs Splicesitefinder (SSF), Alternative Splice Site Predictor (ASSP), NetGene2 v2.4, and Human Splicing Finder splice site analysis (HSF) were used to check if the exon changes affected splice-enhancing sequences, and if the intron changes happened in donor or acceptor splicing sites.

#### Predicting Damaging Amino Acid Substitution

The substitution is predicted to affect protein function with online programs like *Poly*morphism *Pheno*typing (Polyphen), PMUT, and Sorting Intolerant From Tolerant (SIFT).

### (iv) Genotype-Phenotype Correlation

A descriptive study of the phenotypical features of the mutation carriers was undertaken. These characteristics were compared with those of relatives without the mutation in order to study cosegregation and penetrance; taking into account both sex and age. Furthermore, features of the carriers were compared with those in cases with identical mutations previously described in medical literature.

## Results

**Figure 1:**
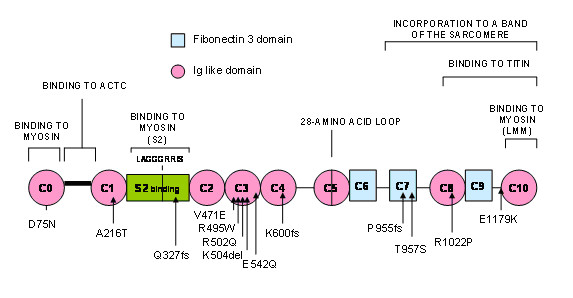
**Myosin binding protein C secondary structure and localization of exonic mutations linked to HCM**.

Sixteen mutations in *MyBPC3 *were identified in 20 of the 130 index cases (15%). Thirteen mutations located in exonic regions were concentrated in 8 motives (Figure 1). Seven of them in, or near, motives implicated in myosin and/or titin binding (C0, C1, S2 binding, C7, C8 and linker C9-C10) and the rest of them in motives in which it is unknown if they establish interactions with other proteins. Three mutations (IVS6+5G>A, IVS11-9G>A and IVS29+5G>A) were located in flanking intronic regions and according to the bioinformatics can affect aberrant transcriptions.

Five of the identified mutations were novel whereas the other eleven mutations had been previously described [11-18] (Table 1). All mutations appeared in heterozygousis, except for IVS6+5G>A, which showed up in homozygousis as we have previously reported [19]. None of the patients with mutations in *MyBPC3 *showed mutations in *MYH7*.

**Table 1:** Mutations in *MyBPC3 *gene.

A. Missense mutations in the *MyBPC3 *gene						
**Fam.**	**Type**	**Mutation**	**Exon/** **Intron**	**Ref seq 2920822**	**Pathogenicity**	

H73	Missense	D75N*	E2	g 2374 G>A	likely	
H42	Missense	A216T[12]	E5	g3898G>A	uncertain	
H279	Missense	V471E*	E16	g10774T>A	likely	
H161	Missense	R495W[13]	E17	g10930C>T	likely	
H614, H147	Missense	R502Q[11]	E17	g10952G>A	likely	
H153, H641, H166	Missense	E542Q[14]	E17	g11071G>C	uncertain	
H120	Missense	T957S[15]	E27	g18572C>G	uncertain	
H49, H18	Missense	R1022P[16]	E29	g19966G>C	likely	
H95	Missense	E1179K[17]	E32	g20989G>A	uncertain	
**B. Deletions in the *MyBPC3 *gene**						

**Fam.**	**Type**	**Mutation**	**Exon/** **Intron**	**Ref seq 2920822**	**Possible effect**	
					**Transcript**	**Traduction**

H13	Deletion	Q327fs*	E12	g7364delG	Frameshift	Truncation (X349)
H46	Deletion	K504del[18]	E17	g10957-9delAAG	In frame	One lost aa (X1273)
H37	Deletion	K600fs[18]	E19	g12413delA	Frameshift	Truncation (X601)
H160	Deletion	P955fs[11]	E27	g18566-7delCT	Frameshift	Truncation (X1049)
**C. Splice mutations in the *MyBPC3 *gene**						

**Fam.**	**Type**	**Mutation**	**Exon/** **Intron**	**Ref seq 2920822**	**Splice site prediction**	

H56	Splicing	IVS6+5G>A*	I6	g5261G>A	splice error	
H110	Splicing	IVS11-9G>A*	I11	g7301G>A	splice error	
H131	Splicing	IVS29+5G>A^#^	I29	g20096G>A	splice error	

A: Missense mutations, B: Deletions and C: Splice mutations.

* Not previously described. ^#^unpublished abstract (Yu et al., HUGO's 10^th ^Human Genome Meeting, Poster 279). Fam: Family; E exon; I intron; aa: aminoacid. In missense mutations, at least two ("likely") or less than two ("uncertain") online programs (Polyphen, PMUT, SIFT) predicted a damaging effect. Splice site prediction was assessed by online programs (SSF, ASSP, NetGene2, HSF).

Three of the sixteen identified mutations were found in more than one family (Table 1). R502Q and R1022P showed up in two families, respectively, and E542Q appeared in three of them.

### (i) Comparative between the phenotype of individuals with mutation: *MyBPC3 vs. MYH7*

There were no significant differences in maximum thickness among index cases with mutations in *MyBPC3 *(25(7) mm) and *MYH7 *(27(8) mm), p = 0.16); and age at time of diagnosis was similar (46(16) vs. 44(19) p = 0.9) (Table 2).

**Table 2:** Phenotypic characteristics of the index patients.

	Without mutation (n = 97)	Mutation *MyBPC3* (n = 20)	Mutation *MYH7* (n = 13)	P (*MyBPC3 vs MYH7*)	P (with *vs *without mutation)
**Age at diagnosis (years)**	53 (16)	46 (16)	44 (19)	0.97	0.051
**Age start follow up (years)**	56 (16)	49(15)	50 (18)	0.86	**0.049**
**Males**	68%	55%	38%	0.46	0.054
**Family history of HCM**	23%	30%	62%	0.25	0.10
**Family history of sudden death**	16%	15%	31%	0.21	0.42
**High blood pressure**	43%	40%	23%	1	0.42
**NYHA initial III-IV**	10.5%	10%	8%	0.60	0.74
**NYHA III-IV ever**	38%	50%	46%	1	0,21
**Angina**	60%	70%	41%	0.45	0.68
**Syncope**	19%	15%	27%	0.64	1
**Non-sustained ventricular tachycardia**	25.3%	42.1%	40%	1	0.10
**Abnormal blood pressure response**	10.8%	31.6%	67%	0.9	**0.030**
**Maximum left ventricular wall thickness(mm)**	22 (6)	25 (7)	27 (8)	0.16	**0.001**
**Wall thickness ≥ 30 mm**	13%	15%	39%	0.95	0.09
**Shortening fraction (%)**	39 (10)	39 (11)	43 (10)	0.29	0.67
**Left atrial diameter (mm)**	45 (7)	50 (11)	50 (14)	0.79	**0.038**
**Gradient ≥ 30 mmHg**	29%	35%	31%	1	0.89

Phenotypic characteristics of the index patients with mutations in *MYH7*, *MyBPC3 *and without mutations in those genes.

HCM: hypertrophic cardiomyopathy; NYHA: New York Heart Asociation functional class

### (ii) Comparative between the phenotype of individuals with and without mutation

There were significant differences in age at time of diagnosis, abnormal blood pressure response, in maximum left ventricular wall thickness and left atrial diameter between patients with mutation (in *MYH7 *or *MyBPC3*) and patients without mutation (Table 2).

Index cases, with mutation were diagnosed younger (p = 0.049), had larger abnormal blood pressure response (p = 0.030), larger left ventricular wall thickness (p = 0.001) and larger left atrial diameter (p = 0.038).

Index cases with mutation had larger frequency of familial history and non-sustained ventricular tachycardia than index cases without mutation, although these differences were not significant.

### (iii) Familial genetic study

A genetic study was carried out in 46 positive index cases relatives: 24 were carriers and 22 non-carriers. Out of those 24 carriers, 14 showed HCM, 4 with suggestive electrocardiographic alterations but not HCM diagnostic (H73-II:6 = D75N H197-III:4 = R502Q, H76-II:7 = K504del and H160-IV:2 = P955fs,)], and 6 women, ages 25 (H279-III:2 = V471E), 29 (H95-III:6 = E1179K), 32 (H95-III:2 = E1179K), 33(H131-IV:4 = IVS29+5G>A), 49 (H46-III:4 = K504del) and 68 (H73-II:7 = D75N), were healthy carriers. Within those 22 non-carriers, 18 were considered healthy, 1 with sugestive electrocardiographic alterations (H73-II:3 = D75N), 2 probably affected (H49-IV:1 & IV:5 = R1022P) and 1 case presented HCM (H153-II:1 = E542Q).

SD as the most serious adverse event was prevalent in 5 of the 20 positive index cases. No genetic test was available for these cases. SDs occurred in 2 of 3 families with splice mutations (H56 = IV6+5G>A, H131 = IVS29+5G>A,), in 1 of 3 with frameshift mutations (H160 = P955fs) and in 2 of 9 with missense mutations (H42 = A216T, H166 = E542Q).

Eight individuals of five families suffered from SD between the ages of 15 and 51.

**Figure 2:**
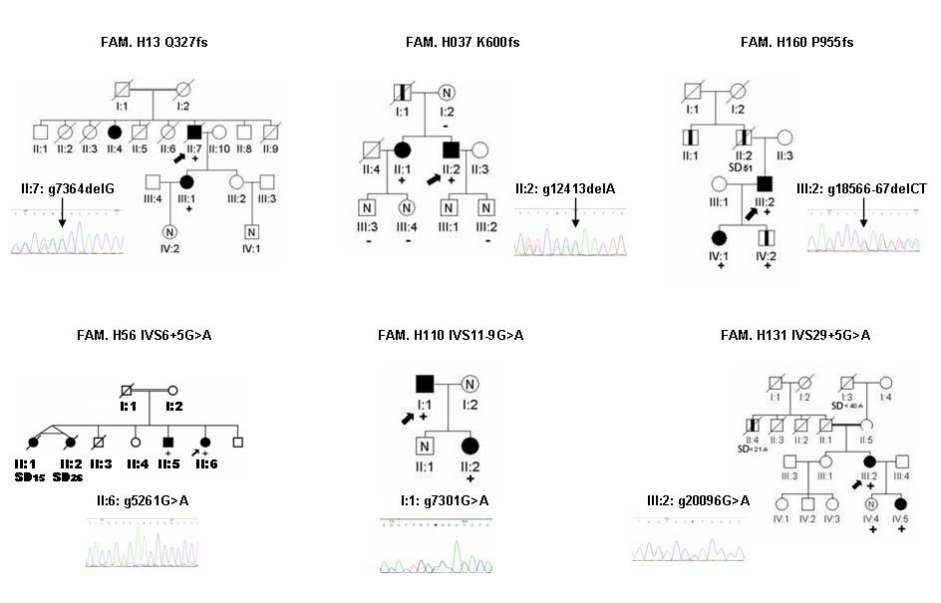
**Pedigrees with the electropherogram of the frameshift and intronic mutations**. Squares are males and circles are females. Filled in black are cases with HCM. Symbols with a black vertical bar represent relatives that were considered possibly affected. N means normal phenotype, empty symbols are non-evaluated relatives. The arrows indicate the index patients. Diagonal lines indicate deceased individual. + indicate carriers of the mutation and - indicates the non-carriers. SD means sudden death.

**Figure 3:**
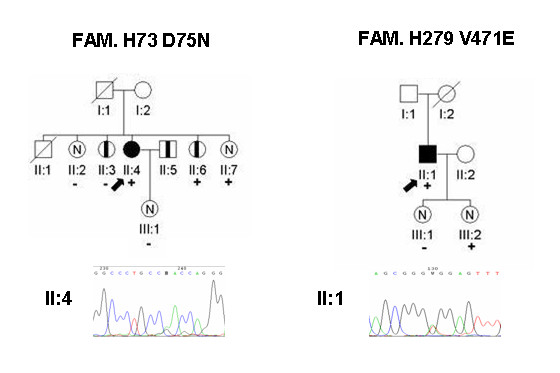
**Pedigrees with the electropherogram of the new missense mutations**. Squares are males and circles are females. Filled in black are cases with HCM. Symbols with a black vertical bar represent relatives that were considered possibly affected. N means normal phenotype, empty symbols are non-evaluated relatives. The arrows indicate the index patients. Diagonal lines indicate deceased individual. + indicate carriers of the mutation and - indicates the non-carriers.

Clinical data of the index cases and carriers can be found in Additional file 1. Figure 2 and figure 3 showed the pedigree of frameshift and splice mutations, and new missense mutations, respectively.

## Discussion

This study has allowed us to identify mutation in *MyBPC3 *in 15% of our index cases, whereas in *MYH7 *were 8% in the same cohort [10]. As in other previous studies, the *MyBPC3 *mutations are more frequently a cause of HCM than mutations in *MYH7 *[18,20-23].

In our study, no family presented two mutations in *MyBPC3 *or in *MyBPC3*+*MYH7*. However, previously we have described in this cohort a family with two mutations (R787H and I736T) in *MYH7 *[10]. This provides us a 0.8% of multiple mutation cases, a lower percentage than the described in the literature (≈ 3%) [18,24,25]. As looking for additional mutations, we also tested all patients, using a genotyping platform which included 537 known mutations in other HCM disease genes (*TNNT2, TNNI3, TPM1, MYL2, MYL3, ACTC, TTN, MYH6, MYLK2, MYO6, TCAP*). None of the index cases in this study had additional mutations in these genes. However, it is possible that there might be new mutations in these genes or mutations in other genes.

The percentages described in this study, 15% for *MyBPC3 *mutations and 0.8% for multiple mutations, were lower than the described in the literature. The high percentage described could be due to polymorphic variants considered as mutations and the inclusion of related probands. In our study, we considered a variation as a mutation if it was not present in 200 unrelated healthy individuals and our index cases were unrelated.

We have not found significant differences between phenotypes of *MyBPC3 *and *MYH7 *carriers, as it has been previously described [24].

### (i) Not previously described mutations

The pathogenicity of the new mutations must be established based upon criteria of cosegregation, absence in health controls, conservation in the evolution and planned functional alteration.

Pathogenicity is very likely in mutations to provoke the introduction of premature stop codons [26,27], including mutations IVS6+5G>A, IVS11-9G>A, and Q327fs (Table 1 and Figure 2). These truncation mutations could affect protein binding to the thick filament and they could alter the sarcomere's structure and function. In fact, these mutations have been associated to the most severe manifestations of HCM and their association with the disease has been confirmed in animal models [28-30]. However, functional studies of these mutations will be essential to elucidate if they act through a dominant-negative mechanism, if the resulting protein continues to be incorporated into the A-band, or through haploinsufficiency, if the enhanced proteolysis of the truncated protein rather alters the stoichiometry of sarcomeric proteins [31].

Pathogenicity is more difficult to confirm in new mutations of the missense type (D75N and V471E). These mutations did not show up in 200 healthy controls and they affected conserved residue. Moreover, the use of *in silico *tools to predict the pathogenicity of missense mutations showed that D75N and V471E were "likely" to be pathological (at least two online programs predicted a damaging effect, Table 1). However, the number of identified carriers is scarce and there are several cases of healthy carriers. The presence of healthy carriers is frequent in the mutations in *MyBPC3 *due to they can have incomplete penetrance and late development in the hypertrophy [11]. To explain these cases we can speculate that, in the case of V471E, one young healthy women carriers could have had a late onset of the disease expression, and in the case of D75N, as the index cases had been diagnosed at the age of 66, the healthy 68 women could develope HCM in the following years. In favour of their relationship with the disease, we have not identified relatives with HCM who do not carry the mutations. Unless, a relative of D75N index case was probably affected but does not have the mutation; this fact could mean that another factor could be contributing to the development of the HCM.

Therefore, in order to establish the pathogenicity of new mutation, more data about affected and non-affected relatives is needed, and this is not always possible, as in the cases of dead subjects and in those who have declined to participate.

### (ii) Previously described mutations

The identification of a known mutation is clinically more useful, because previous information allows for a better evaluation of the pathogenicity and the genotype-phenotype correlation. For example, the familial study in mutation K504del does not let us confirm its pathogenicity (out of four carriers, one is healthy and another one is questionable, Additional file 1), but the existence of 2 previously described families [18,21] supports the association of the mutation with the disease. In the same way, one of the families with E542Q mutation (H153) has a member who is probably affected and who does not have the mutation. The existence of multiple families in our study (H166, H641) and in other nine families described in the literature [14,18,22-24,32-34] allows us to confirm its association with the disease and it forces us to review its diagnosis (the light-moderate hypertrophy in the electrocardiogram must be confirmed).

Whereas, in mutation R1022P, two possible HCM patients do not have the mutation, and in the case previously described in the literature there are no data about the familial study [16], so it is not possible to verify its pathogenicity.

On the other hand we provide useful information for the clinicians, like in mutation K600fs (Figure 2), our data confirms the pathogenicity of a previously described mutation in just one case without available clinical data or familial study [18].

### (iii) Severe and early forms of HCM and SD in patients with mutations in *MyBPC3*

It has been suggested that mutations in gene *MyBPC3 *are associated with a light clinical and morphological expression and with a late development of hypertrophy [11,35]. However, we identify a relevant number of cases with early severe hypertrophy development, as in the cases of carriers of the following mutations: R502Q, a post-myectomy 42 year old with 20 mm and another 36 mm case at 15; and P955fs, a 28 mm case at 36 and a 16 year old female with 21 mm. Moreover, we also have cases with atrial fibrillation at a young age (K600fs, R1022P, IVS6+5G>A) and several cases of people who underwent surgery when they were young (R502Q and IVS6+5G>A). Nevertheless, when we gather data about our families and those from previous studies [11,14,16,18,21-24,32-35] (Table 3), we see that in several mutations (R502Q, E542Q, K600fs, P955fs) average age at time of diagnosis is not advanced and thickness is above average in HCM.

**Table 3:** Genotype-phenotype correlation in previously described mutations.

Mutation	R502Q	K504del	E542Q	K600fs	P955fs	R1022P
**No. of carriers (HCM/healthy)**	23 (17/6)	6 (5/1)	18 (16/0)	3 (3/0)	19 (15/4)	6 (6/0)
**No. of non-carriers (HCM/healthy)**	10 (0/10)	2 (0/2)	11 (1/10)	3 (0/3)	9 (0/9)	5 (2/3)
**No. of controls**	350	450	550	200	400	100
**Mean age at HCM diagnosis (range)**	44 (15-81)	38 (21-59)	45 (16-53)	47 (44-50)	25 (16-36)	42 (23-67)
**Mean maximal wall thickness (range)**	22 (10-37)	19 (9-34)	23 (17-34)	22 (19-25)	23 (8-35)	18 (14-28)
**No. of sudden deaths/No. of families**	1/7	0/3	2/12	0/2	4/4	0/3
**Other events**	-	2 CVA deaths	1 CVA death	2 CVA (1 death)	-	-
**References**	[11,21,23,35]	[18,21]	[14,18,22-24,32-34]	[18]	[11,18,24]	[16]

CVA: cerebrovascular accident. HCM: hypertrophic cardiomyopathy.

HCM has been recognized as the most common cause of SD in the young, especially in competitive athletes [1]. The families associated with SD presented missense mutations (A216T, E542Q) and mutations that lead to aberrant transcripts (P955fs, IVS6+5G>A, IVS29+5G>A).

The association between SD and missense mutations A216T and E542Q is controversial; probably there are other additional factors that can be interacting. The index case of A216T was a woman with HCM and thrombotic problems (she needed two surgical aortic valve replacements and finally went into heart transplantation). On the other hand, the E542Q mutation was present in 3 of our 130 families, but only in one of them has cases of SD.

In our families the most strong association of SD with the mutations is in the mutations that lead to protein truncation (P955fs, IVS6+5G>A, IVS29+5G>A). In fact, the youngest SD (15 and 26 year old) are in the IVS6+5G>A index cases families, and these individuals are likely to be in homozygousis as in the index case. This fact could support a gene dossage effect for mutations in the *MyBPC3 *gene that have been previously described where a homozygous mutation is associated with a more severe phenotype than the heterozygous [19].

## Conclusions

We have identified mutations in *MyBPC3 *in 15% of our families with HCM. Severe hypertrophy and an early expression of the disease are compatible with the presence of mutations in *MyBPC3*.

Genetic studies can play a key role in the comprehensive evaluation of familiar hypertrophic cardiomyopathy and in the development of individualized medicine. This kind of analysis not only allows avoiding clinical follow up of non carriers but it opens new possibilities including: taking preventive clinical decisions in mutation carriers than have not developed the disease yet, the establishment of genotype-phenotype relationship, and establishing a genetic diagnosis routine in patients with familial HCM.

To obtain these purposes, it is important to perform the genetic analysis of index cases and in a high number of their relatives. It is necessary to obtain detailed data about the phenotype of a larger number of carriers, and to complete the genetic study in the severe cases through analysis of other genes in order to establish in which degree the severity of the phenotypes is a feature of the identified mutations or if it depends upon additional factors.

## Competing interests

The authors declare that they have no competing interests.

## Authors' contributions

LM and MHP conceived and designed the study. MIRG carried out the molecular genetic studies, participated in the sequence alignment and drafted the manuscript. LC, LN and EM participated in the sequence alignment. LM, MO, XF, RBV and EV acquired and interpreted the clinical data. ACB handled funding and supervised the study. Finally, MIRG, LM, LN and MHP drafted the manuscript. All authors read and approved the final manuscript.

## Pre-publication history

The pre-publication history for this paper can be accessed here:

http://www.biomedcentral.com/1471-2350/11/67/prepub

## Supplementary Material

Additional file 1 **Clinical characteristics of *MyBPC3 *mutation carriers and affected non-carriers**. In this table, it is shown the clinical characteristics of *MyBPC3 *mutation carriers and affected non-carriers. Click here for file
