# Supplementary material for: Screening mutations in myosin binding protein C3 gene in a cohort of patients with Hypertrophic Cardiomyopathy
Source: BMC Med Genet. 2010 Apr 30;11:67. doi: 10.1186/1471-2350-11-67 (PMC2880974; doi:10.1186/1471-2350-11-67)
Supplement: Additional file 1 — Clinical characteristics of MyBPC3 mutation carriers and affected non-carriers. In this table, it is shown the clinical characteristics of MyBPC3 mutation carriers and affected non-carriers. [file 1471-2350-11-67-S1.DOC]

**Additional file 1- Clinical characteristics of *MyBPC3* mutation carriers and affected non-carriers.**

| **Mutation** | **Fam.** | **Case** | **Mut** | **Sex** | **Age diagnosis** | **Age FU** | **WT (mm)** | **S** | **SO** | **ABPR** | **NSVT** | **FHSD** | **NYHA** | **ECG** | **Intervention** | **Event** |
| --- | --- | --- | --- | --- | --- | --- | --- | --- | --- | --- | --- | --- | --- | --- | --- | --- |
| **D75N** | H73 | II:3 | N | F | NoDx | 77 | 13 | + | - | ¿ | ¿ | - | I | LBBB | - | - |
|  |  | II:4* | Y | F | 66 | 72 | 18 | - | - | - | - | - | II | T (-) | - | - |
|  |  | II:6 | Y | F | No Dx | 70 | 12 | - | - | ¿ | ¿ | - | II | LBBB | - | - |
|  |  | II:7 | Y | F | No Dx | 68 | - | - | - | ¿ | ¿ | - | I | ¿ | - | - |
| **A216T** | H42 | II:6* | Y | F | 34 | 41 | 25 | - | +++ | + | - | + | III-IV | AF-LVH | SA, ICD, MP | TX |
| **IVS6+5G>A** | H56 | II:5 | Y | M | 28 | 40 | 26 | - | - | ¿ | + | + | II | AF-IRBBB | - | - |
|  |  | II:6* | Y | F | 34 | 39 | 24 | - | - | + | + | + | II | T(-) | ICD | AD |
| **IVS11-9G>A** | H110 | I:1* | Y | M | 58 | 62 | 17 | - | + | - | - | - | I | Normal | - | - |
|  |  | II:2 | Y | F | 21 | 27 | 17 | - | - | - | - | - | I | LVH, T(-), LABBH | - | - |
| **Q327fs** | H13 | II:7* | Y | M | 64 | 73 | 19 | + | - | ¿ | + | - | III | AF, Q | + | Death CHF |
|  |  | III:1 | Y | F | 37 | 37 | 16 | - | - | ¿ | ¿ | - | I | Normal | - | - |
| **V471E** | H279 | II:1* | Y | M | 57 | 65 | 17 | - | - | - | - | - | I | LVH, T(-) | - | - |
|  |  | III:2 | Y | F | No Dx | 25 | 9 | - | - | - | - | - | I | Normal | - | - |
| **R495W** | H161 | II:4* | Y | M | 71 | 75 | 18 | - | - | - | + | - | I | AF, LVH, T(-) | - | - |
| **R502Q** | H147 | II:2 | Y | M | 50 | 70 | 23 | + | - | + | + | - | II | LVH, T(-) | - | - |
|  |  | II:4* | Y | M | 42 | 66 | 20 | - | - | - | - | - | II | T(-) | Myectomy, PM | - |
|  |  | III:4 | Y | M | No Dx | 34 | 11 | - | - | - | - | - | I | Q | - | - |
|  | H614 | I:1 | Y | M | 68 | 69 | 17 | - | - | - | - | - | I | Normal | - | - |
|  |  | II:3* | Y | M | 15 | 36 | 36 | + | ++ | + | + | - | I | LVH, Q | ICD | - |
| **K504del** | H46 | II:7 | Y | M | ¿ | ¿ | ¿ | ¿ | ¿ | ¿ | ¿ | ¿ | ¿ | ¿ | - | - |
|  |  | II:8 | Y | M | 59 | 76 | 17 | - | - | + | - | - | II | Q, PM | PM | - |
|  |  | III:1* | Y | M | 35 | 52 | 19 | - | - | - | - | - | I | Q, LVH | - | - |
|  |  | III:4 | Y | F | No Dx | 49 | 9 | - | - | - | - | - | I | Normal | - | - |
| **E542Q** | H153 | II-1 | no | M | 58 | 70 | 17 | - | - | - | - | - | I | N | - | ACV(70) |
|  |  | II:4* | Y | F | 44 | 51 | 20 | - | +++ | + | - | - | II | LVH, T(-) | MP | - |
|  |  | III:3 | Y | M | 16 | 17 | 14 | - | - | - | - | - | I | LVH | - | - |
|  | H166 | III:7* | Y | F | 53 | 61 | 23 | - | - | - | - | + | III | AF | PM | - |
|  | H641 | II:2* | Y | F | 65 | 73 | 28 | - | - | - | - | - | II | AF, Q, ST | - | - |
| **K600fs** | H37 | II:1 | Y | F | 50 | 59 | 21 | + | - | + | ¿ | - | II | AF-LBBB | PM | - |
|  |  | II:2* | Y | M | 44 | 58 | 23 | - | ++ | + | - | - | II | AF, Q T(-) | - | FE |
| **P955fs** | H160 | III:2* | Y | M | 36 | 49 | 28 | - | - | - | - | + | I | LVH, Q, T(-) | - | - |
|  |  | IV:1 | Y | F | 16 | 28 | 21 | - | - | - | - | + | I | LVH, T(-) | - | - |
|  |  | IV:2 | Y | M | No Dx | 23 | 8 | - | - | - | - | + | I | Q | - | - |
| **T957S** | H120 | II:3* | Y | M | 44 | 56 | 12 | - | - | - | - | - | II | LVHA, | - | - |
| **R1022P** | H18 | II:5* | Y | M | 52 | 76 | 20 | - | - | - | + | - | I | AF, LVH, Q, ST | - | - |
|  | H49 | II:2 | Y | F | 67 | 67 | 15 | - | - | ¿ | ¿ | - | II | LBBB | - | - |
|  |  | III:2 | Y | F | 21 | 46 | 14 | + | - | - | + | - | II | AF, Q, RBBB | - | - |
|  |  | III:4 | Y | M | 44 | 45 | 24 | - | - | ¿ | ¿ | - | I | T(-) | - | - |
|  |  | III:5* | Y | F | 27 | 41 | 16 | - | - | - | - | - | I | LVH, Q | - | - |
|  |  | IV-1 | N | M | 22 | 29 | 12 | - | - | - | - | - | I | HVI  Q | - | - |
|  |  | IV-5 | N | M | 17 | 22 | 13 | - | - | - | - | - | I | HVI  Q | - | - |
| **IVS29+5G>A** | H131 | III:2* | Y | F | 45 | 54 | 41 | - | + | - | + | + | III | LVH, T(-) | - | - |
|  |  | IV:4 | Y | F | No Dx | 33 | 10 | - | - | ¿ | ¿ | + | I | Normal | - | - |
|  |  | IV:5 | Y | F | 24 | 31 | 15 | - | - | ¿ | - | + | I | Normal | - | - |
| **E1179K** | H95 | II:1* | Y | M | 64 | 69 | 22 | - | - | - | - | - | II | AF, LVH,  T(-), Q | - | Ictus (66) |
|  |  | II:4 | Y | F | 63 | 67 | 19 | - | + | - | - | - | II | LVH | - | - |
|  |  | III:2 | Y | F | No Dx | 32 | 8 | - | - | ¿ | ¿ | - | I | Normal | - | - |
|  |  | III:6 | Y | F | No Dx | 29 | 7 | - | - | - | - | - | I | Normal | - | - |

ABPR: Abnormal blood pressure response, AD: Appropriate discharge, AF: atrial fibrillation, Age FU: Age last follow up, FE: Femoral embolism, FHSD: family history of sudden death, RBBB: right bundle branch block, LBBB: left bundle branch block, ICD: implantable cardioverter defibrillator, ECG: electrocardiogram, Fam: family, LABBH: left anterior bundle branch hemiblockade, LVH: left ventricular hypertrophy, CHF: congestive heart failure, PM: pacemaker, MP: Mitral prothesis, Mut: presence of mutation, N: no, No Dx: Unaffected or healthy, NYHA: New York Association functional class, Q: abnormal Q wave, S: Syncope, SA: Septal ablation, ST: ST segment abnormalities, SO: Subaortic obstruction , T(-): negative T wave, NSVT: non-sustained ventricular tachycardia, TX: cardiac transplantation, WT: Max wall thickness, Y: yes, *: index case.
